# Inhibitory control and decimal number comparison in school-aged children

**DOI:** 10.1371/journal.pone.0188276

**Published:** 2017-11-20

**Authors:** Margot Roell, Arnaud Viarouge, Olivier Houdé, Grégoire Borst

**Affiliations:** 1 Université Paris Descartes, Université Sorbonne Paris Cité, Laboratoire Psychologie du Développement et de l’Éducation de l’Enfant (LaPsyDÉ), UMR CNRS 8240, Paris, France; 2 Université de Caen, Caen, France; 3 Ecole des Neurosciences de Paris (ENP), Paris, France; 4 Institut Universitaire de France, Paris, France; Katholieke Universiteit Leuven, BELGIUM

## Abstract

School-aged children erroneously think that 1.45 is larger 1.5 because 45 is larger than 5. Using a negative priming paradigm, we investigated whether the ability to compare the magnitude of decimal numbers in the context in which the smallest number has the greatest number of digits after the decimal point (1.45 vs. 1.5) is rooted in part on the ability to inhibit the “greater the number of digits the greater its magnitude” misconception derived from a property of whole numbers. In Experiment 1, we found a typical negative priming effect with 7^th^ graders requiring more time to compare decimal numbers in which the largest number has the greatest number of digits after the decimal point (1.65 vs. 1.5) after comparing decimal numbers in which the smallest number has the greatest number of digits after the decimal point (1.45 vs. 1.5) than after comparing decimal numbers with the same number of digits after the decimal point (1.5 vs. 1.6). In Experiment 2, we found a negative priming effect when decimal numbers preceded items in which 7^th^ graders had to compare the length of two lines. Taken together our results suggest that the ability to compare decimal numbers in which the smallest number has the greatest number of digits is rooted in part on the ability to inhibit the “greater the number of digits the greater its magnitude” misconception and in part on the ability to inhibit the length of the decimal number per se.

## Introduction

Understanding of decimal numbers is crucial for subsequent academic and occupational success [1]. However, according to a nationally representative sample of 1,000 US mathematics teachers, poor knowledge of “rational numbers and operations involving fractions and decimals” is one of the two greatest obstacles preventing their students from learning algebra [2]. Indeed, although students learn about decimals in primary schools, secondary students use decimals without an adequate knowledge of the concepts involved [3,4,5]. Understanding of decimal numbers remains weak even in students of countries that are top performers on international comparison of mathematical achievement such as China [6]. Notably, students lack the sufficient understanding of the comparative size of decimal numbers [7]. Difficulty in decimal comparison has been documented not only in children throughout schooling [8,9,10] but also in adults [11,12].

Decimal comparison appears to be particularly difficult when the decimal numbers to compare do not have the same number of decimal places [13]. In this context, children tend to erroneously think that 1.45 is larger than 1.5 because 45 is larger than 5. These errors are likely the result of a “whole number” bias in this specific case, using a property of whole numbers such as “the greater the number of digits the greater its magnitude” to compare decimal numbers in which the smallest one has the greatest number of digits after the decimal point [12,14,15, 16].

Indeed, while a competent understanding of whole numbers is crucial to the development of mathematical understanding, it may also interfere in mathematical reasoning when rational numbers are involved [15]. Learners may assume implicitly or explicitly that the features of whole numbers continue to apply to rational numbers, inducing systematic errors when rational numbers behave differently from whole numbers [4,16,17]. According to Vosniadou and colleagues [16,18,19], during the preschool years, children form an initial concept of numbers. This concept, based on whole numbers, encompasses assumptions, beliefs, and expectations of what counts as a number and how it is supposed to behave. As rational number information violates basic principle of the whole number concept, children must restructure the whole number concept and construct a new representation of rational numbers. This new representation does not however replace the initial representation of whole numbers but rather co-exists with it [20]. For instance, individuals commit more errors and require more time to compare decimal numbers in items in which the whole number properties interfere with the decimal number properties (i.e., incongruent items such as 1.45 vs. 1.5) than in items in which the whole number properties are congruent with the decimal number properties (i.e., congruent items such as 1.45 vs 1.4) [21].

Van Hoof and colleagues [22,23] also provided evidence for a whole number bias in secondary students processing algebraic expressions (“multiplication and addition always lead to larger outcomes” and “division and subtraction always lead to smaller outcomes”) and comparing fractions (considering that fractions are two (natural) numbers rather than a single number) as suggested by higher accuracy levels on congruent (e.g., x*5 < x; 3 < 3/x) than incongruent (e.g., x < x*4; x/5 < x) items when processing algebraic expressions as well as longer reaction times when comparing fractions in incongruent items (e.g., x/a vs. x/b) than on congruent ones (e.g., a/x vs. b/x).

Finally, Durkin and Rittle-Johnson [24], in a longitudinal study assessing knowledge and diagnosing misconceptions about decimals in 4th and 5th graders, found that the “greater the number of digits the greater its magnitude” misconception was one of the most common misconceptions when comparing decimal numbers, and that this misconception decreased over time. Studies so far have focused on defining the context in which decimal comparison errors occur and the misconceptions that might be at the root of these errors. However, the mechanisms allowing children to overcome such errors remain largely unknown.

In light of the fact that executive functions and inhibitory control in particular (i.e., the ability to resist habits, automatisms and misconceptions [25]) is one of the core mechanisms of cognitive development [26,27,28,29,30,31] and mathematical development in particular [21,32,33,34,35,36,37,38], we hypothesized that inhibitory control may be one of the mechanisms allowing children to overcome systematic errors when comparing decimal numbers such as 1.45 and 1.5. Indeed, Bull and Lee [39] in their narrative review highlight different ways in which inhibition may influence math achievement: inhibition may suppress the use of information from a word problem that is irrelevant to the solution (e.g., “add if more or subtract if less” [40]), it may supress inappropriate strategies (e.g., addition when subtraction is needed) or proponent number representation (e.g., whole number biases).

This assumption is consistent with the dual-process theory of human reasoning according to which systematic reasoning errors (or reasoning biases such as the whole number bias) in different domains may be in part related to our tendency to rely on heuristics (i.e. fast automatic and holistic strategies such as the “greater the number of digits the greater its magnitude” strategy) in contexts in which we should we should rely on algorithmic strategies (i.e. slow cognitively demanding and analytical strategies) [41,42]. To correctly respond to situations where algorithmic strategies are in conflict with heuristic strategies, studies have provided convergent evidence that inhibitory control is necessary to avoid using a misleading heuristic strategy [43,44,45].

Thus, in the present study, we investigated in Experiment 1 whether children’s ability to compare the magnitude of a pair of decimal numbers whereby the smallest one has the greatest number of digits after the decimal point (e.g., 1.45 vs. 1.5) is rooted in the ability to inhibit the “greater the number of digits the greater its magnitude” misconception. In Experiment 2, we aimed to determine whether in this context, the length of the decimal number (i.e. the spatial extent of the number in a spatial continuous magnitude sense) per se would also need to be inhibited too.

In both experiments, we used a negative priming paradigm to determine whether inhibition may be required. The negative priming paradigm rests on the logic that if a strategy (or a misconception) is inhibited on a given item, then the activation of that strategy (or misconception) on the next item should be more difficult as revealed by poorer performance [46, 47]. Using a negative priming approach, studies have provided evidence for the role of inhibitory control in overcoming systematic errors in the resolution of arithmetic word problems in children, adults and experts [37,40], in quantitative reasoning in geometry [26], in logical reasoning about class inclusion [48,49] and number conservation [50], as well as in the understanding of the physical principle governing the flotation of objects [51]. Note that the negative priming approach requires that participants can perform above chance level for the prime items. Since pre-tests revealed that children before grade 7 were at chance level of performance when comparing the magnitude of decimal numbers in which the smallest decimal number has the greatest number of digits after the decimal point (e.g., 4.5 vs 4.233), we included 7^th^ graders in our study.

## Experiment 1

We used a negative priming paradigm to determine whether the “greater the number of digits the greater its magnitude” misconception must be inhibited to compare the magnitude of decimal numbers in which the smallest number has the greatest number of digits after the decimal point. We designed a negative priming paradigm in which for both prime and probe items, children were to compare decimal numbers and identify the largest. In the test condition, congruent probe items, in which the largest number had the greatest number of digits after the decimal point (e.g., 7.899 vs. 7.4, a context that automatically triggers the “greater the number of digits the greater its magnitude” misconception, thus helping to determine the largest decimal number) were preceded by incongruent prime items (i.e., the prime) in which the smallest decimal number had the greatest number of digits after the decimal point (e.g., 4.5 vs. 4.233, a context that supposedly requires to inhibit the “greater the number of digits the greater its magnitude” misconception). In the control condition, the same congruent probe items, were preceded by neutral prime items, for which the “greater the number of digits the greater its magnitude” strategy was irrelevant since both decimal numbers had the same number of decimal places (e.g., 8.1 vs 8.5).

We reasoned that if the “greater the number of digits the greater its magnitude” misconception must be inhibited to compare incongruent items, decimal numbers in which the smallest number has the greatest number of digits after the decimal point, then a negative priming effect should be observed: children should be less efficient to determine which of two decimal numbers is the largest in congruent items (e.g., 7.899 vs 7.4) after having compared the magnitude of decimal numbers in an incongruent item (e.g., 4.5 vs 4.233) than after having compared two decimal numbers in a neutral item (e.g., 8.1 vs 8.5).

### Method

#### Participants

We recruited 26 children with an average of 12.4 ± 0.52 years with normal or corrected-to-normal vision from a public high-school serving a diverse population (Paris, France). We excluded two children that scored at chance levels, leading to a group of 24 children (9 males) with an average of 12.35 ± 0.51 years. We obtained informed written consent from parents as well as oral consent from all children. Children were tested in accordance with national and international norms governing the use of human research participants. The Faculty of Psychology (Paris Descartes University) granted the ethical permission to conduct this study.

#### Materials

Stimuli were presented on a laptop computer (resolution of 1366 × 768 pixels and a refresh rate of 60 Hz), using E-Prime 2.0. Prime items consisted of pairs of decimals written in 24-point Courier New font, each located on the left or right side of the screen at a 0.5° visual angle from the centre. In the test condition, prime items were pairs of decimal numbers in which the smallest decimal had the greatest number of digits after the decimal point (e.g., 3.453 vs. 3.6). In the control condition, prime items consisted in pairs of decimal number with the same number of digits after the decimal point (e.g., 7.3 vs. 7.6). In both conditions, probe items consisted of pairs of decimal numbers in which the largest decimal had the greatest number of digits after the decimal point (e.g., 5.644 vs. 5.4). Decimal numbers had either one, two or three digits after the decimal point. Decimals in the pairs had either both the same number of decimal points or a different number of digits after the decimal point. The difficulty of the comparison was systematically varied by manipulating the numerical distance between the first digits after the decimal point of each decimal number in the pair (distance ranged between 1 and 6).

#### Procedure

**Fig 1:**
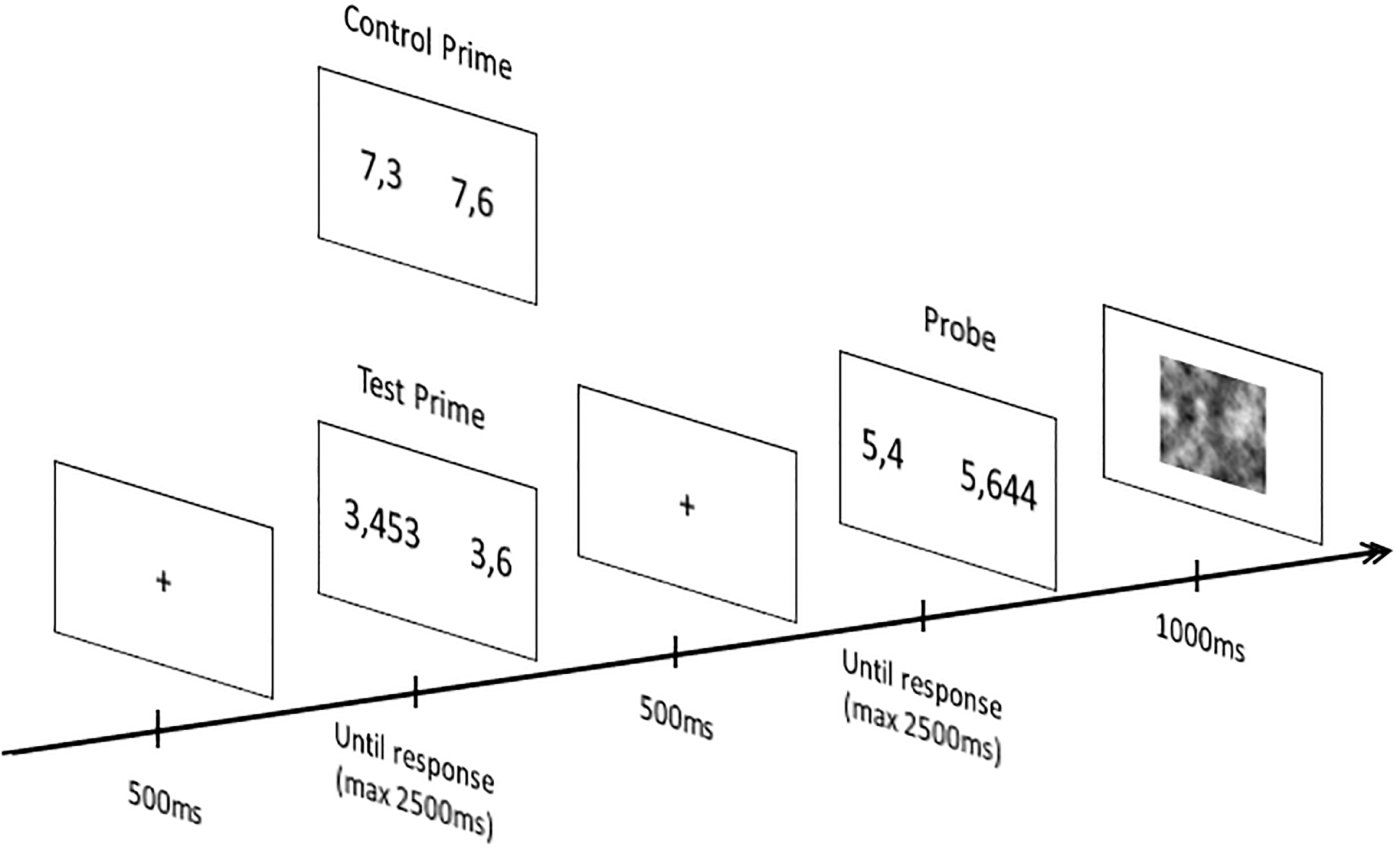
Example of prime and probe items presented in the test and the control conditions. Prime items (i.e., incongruent and neutral items) differed between the two conditions but the probe items (i.e., congruent items) were similar.

Children were tested individually, seating approximately 75cm from the laptop computer. For each pair of decimal numbers presented, children were instructed to determine which of the two decimal numbers was the largest. Children pressed the left button on the mouse to indicate that the decimal number on the left of the screen was the largest and the right button to indicate that the decimal number on the right was the largest. As shown in Fig 1, each trial started with the presentation of a fixation cross (500 ms), then a pair of decimal numbers was displayed (until the children answered with a time limit of 2500 ms). As soon as the children provided an answer the fixation point reappeared (500 ms), followed by another pair of decimal numbers that persisted until children provided an answer (with a time limit of 2500 ms). A visual mask was presented between each trial to avoid the transfer of processes from the probe of one trial to the prime of the next trial (1000 ms). Children first performed a block of 6 practice trials in which they were first presented with 2 congruent items, then 2 neutral items and finally 2 incongruent items with pairs of decimal numbers different from the ones used in the experimental trials. Children received simple feedback (correct/incorrect) on their accuracy during the practice trials. Then, they performed a blocks of 48 trials (24 in the test and 24 in the control condition). All sequences of motor responses between the prime and the probe appeared equally often. The order of the trial was randomized, except no more than three tests or control trials could occur in a row.

### Results

Prime or probe responses times (RTs) less or greater than 2 SD from the individual mean for a given condition were deleted (M = 7 ± 5%). For each child, we averaged the RTs and accuracies separately for control and test primes and probes. Note that in accordance with the logic of the NP paradigm, we only analysed accuracies, RTs and IES on the probe items preceded by prime items performed correctly. We then computed, for each child, the inverse efficiency score (i.e., RTs divided by the proportion of correct answers [52,53]) for the primes and the probes of the control and the test conditions. Note that our data complied with the recommendations of Bruyer and Brysbaert [52] for the use of inverse efficiency score (IES): accuracy was high (i.e., greater than 85%), accuracies and RTs went in the same direction and we observed no speed-accuracy trade-off (*r*s < .26, *p*s *>* .21). The IES enables us to simplify our analysis by integrating accuracy scores and RTs in one variable and by appropriately weighing the impact of speed and accuracy. For each of the analyses, we report the effect size in terms of the difference of the means (Cohen’s d). Two-sided Bayesian paired t-test (with a default Cauchy prior width of r = 0.707) were used to quantify the evidence for (BF_01_) or against (BF_10_) the null hypothesis. Bayesian Analyses were performed using the JASP software (Version 0.8.3.1).

A paired bilateral t-test on the prime accuracies, RTs and IES revealed that children were less efficient to compare the magnitude of two decimal numbers in the incongruent items (e.g., 1.45 vs. 1.5) than in the neutral items (e.g., 7.45 vs. 7.55), t(23) = 4.47, *p* < .001, *d* = .91, BF_10_ = 165 for accuracies, t(23) = 6.34, *p* < .001, *d* = 1.29, BF_10_ = 10575 for RTs and t(23) = 5.30, *p* < .001, *d* = 1.08, BF_10_ = 1050 for IES (see Table 1 and S1 Table).

**Table 1:** Accuracies, RTs and IES in the two types of prime items (incongruent and neutral items) and the two types of congruent probe items (preceded by incongruent or neutral prime items) in Experiment 1. Standard deviations appear in parentheses. Negative priming reflects the difference in performance between the two types of congruent probe items.

	Prime		Probe		
	Incongruent item	Neutral item	Preceded by an incongruent prime item	Preceded by a neutral prime item	Negative Priming
Accuracy (%)	88.8 (9.4)	97.7 (4.0)	98.0 (3.0)	98.0 (2.45)	0 (4.5)
Reaction Time (ms)	1159 (280.9)	1004 (217.6)	1146 (290.3)	1067 (234.7)	79 (104.1)
IES	1322 (384.0)	1026 (215.9)	1170 (300.3)	1087 (235.4)	83 (127.8)

A paired bilateral t-test on the probe RTs and IES revealed a negative priming effect: children were less efficient to compare the magnitude of two decimal numbers in congruent items (5.456 vs 5.4) after comparing a pair of decimal numbers in incongruent items (e.g., 1.45 vs. 1.5) than after comparing a pair of decimal numbers in neutral items (e.g., 7.45 vs. 7.55), t(23) = 3.74, *p* < .001, d = 0.79, BF_10_ = 33 for RTs and t(23) = 3.18, *p* = .004, *d* = 0.64, BF_10_ = 10 for IES. A paired bilateral t-test on the probe accuracies revealed no difference in accuracy between congruent items preceded by incongruent items and congruent items preceded by neutral items, t < 1, BF_01_ = 4, probably due to a ceiling effect (accuracy above 98%) (see Table 1).

### Discussion

Consistent with previous studies [3,12,14,15,16,24] we found that children were less efficient to compare the magnitude of two decimal numbers in incongruent items, when the smallest one had the greatest number of digits after the decimal point (1.5 vs. 1.45) than in neutral items, when the two decimal numbers had the same number of decimal place (1.5 vs. 1.4). Importantly, children were less efficient to determine which of two decimal numbers was the largest in congruent items, when the largest one had the greatest number of digits after the decimal point (1.545 vs. 1.4, i.e., a context in which the “greater the number of digits the greater its magnitude” misconception leads to the correct answer) when preceded by an incongruent item than when preceded by a neutral item. Taken together, our results suggest that children’s ability to compare the magnitude of decimal numbers in a context in which the smallest number has the greatest number of digits after the decimal point is rooted in part in the ability to inhibit the “greater the number of digits the greater its magnitude” misconception. Our finding is in agreement with and complements findings from previous studies showing that executive functions and inhibitory control in particular play an important role in the development of mathematical abilities [21,32,33,34,35,36,37,38].

A limitation of Experiment 1 is the limited number of participants that might have affected the power to detect significant effect between the conditions of interest. Note, however that we did observed significant difference between these conditions (a) with high effect size, *ds >*.*64* and (b) Bayes factors in favour of the alternative hypothesis to the null hypothesis, all BF_10_ > 10.

The “greater the number of digits the greater its magnitude” misconception is generally thought to be a consequence of an overgeneralization of children’s knowledge of whole numbers to decimal numbers [4,20]. In particular, children tend to consider all numbers as discrete quantities and that numbers with more digits are larger [54]. Children presumably generate the “greater the number of digits the greater its magnitude” misconception when they integrate rational numbers to their conception of numbers originally based on whole numbers. In Experiment 2 we investigated whether the difficulty in comparing decimal numbers for which the smallest number has more digits after the decimal place (1.45 vs 1.5) could be also due to the fact that the smallest number (1.45) is longer than the largest one (1.5).

## Experiment 2

Studies on the development of mathematical cognition have provided evidence for the existence of preverbal numerical capacities prior to mathematic education [55]. For instance, new-borns already possess the capacity to estimate approximately the number of objects in a collection (i.e. its numerosity [56,57]). The representation of numerosity is associated with the activation of a population of neurons within the intra-parietal sulcus (IPS) in humans [58] independently of their culture of origin [59,60] and in primates [59].

However, neurons in the IPS are not exclusively activated in response to numerosity processing but also in response to non-symbolic dimensions of magnitude such as length, density and size [61]. Importantly, because of the overlap between the neuronal population coding for numerosity and the one coding for non-symbolic dimensions of magnitude, these non-symbolic dimensions of magnitude tend to interfere with numerical judgment [62,63]. A seminal example of this type of interference can be observed in Piaget’s number-conservation task in which children up to 7 years of age commit systematic errors in judging the relative numerosity of two rows of tokens when they differ in length but not in numerosity [50,64,65]. Because symbolic numerical processing activates similar region of the IPS as the ones activated in response to numerosity processing [66], non-symbolic dimensions of magnitude such as length could potentially interfere with symbolic numerical processing as well. In the case of decimal number processing, the difficulty in comparing decimal numbers for which the smallest number has more digits after the decimal place (1.45 vs 1.5) could thus be due in part to the fact that the smallest number (1.45) is longer than the largest one (1.5).

In Experiment 2, we designed a negative priming paradigm to test the following hypothesis: Seventh graders were asked to compare, on the prime, the magnitude of a pair of decimal numbers and then, on the probe, the length of a pair of lines. In the test condition, an incongruent item, a pair of decimal numbers in which the smallest number has the greatest number of digits (e.g., 7.299 vs 7.4, a context that requires supposedly to inhibit the length of the decimal numbers to process their magnitude), preceded a pair of lines (a context in which magnitude comparison is based on the length of the stimuli). In the control condition, a pair of lines was preceded by a neutral items, a pair of decimal numbers with the same number of decimal places (e.g., 8.1 vs. 8.5, a context in which the two numbers have the same length).

We reasoned that if comparing decimal numbers for which the smallest number has the greatest number of digits requires to inhibit the lengths of the numbers (i.e., the spatial extent of the numbers in a spatial continuous magnitude sense) to process their magnitude, then children should be less efficient to compare the length of two lines when preceded by a pair of decimal numbers in which the smallest number has the greatest number of digits than a pair of decimal numbers with the same number of decimal places.

### Method

#### Participants

We recruited 37 children (M = 12.82 ± 0.92 years, 21 males) with normal or corrected-to-normal vision from the same public high-school as the children recruited in Experiment 1. None of the children participated in Experiment 1. We obtained informed written consent from all institution and parents as well as oral consent from all children. Children were tested in accordance with national and international norms governing the use of human research participants. The Faculty of Psychology (Paris Descartes University) granted the ethical permission to conduct this study.

#### Materials and procedure

**Fig 2:**
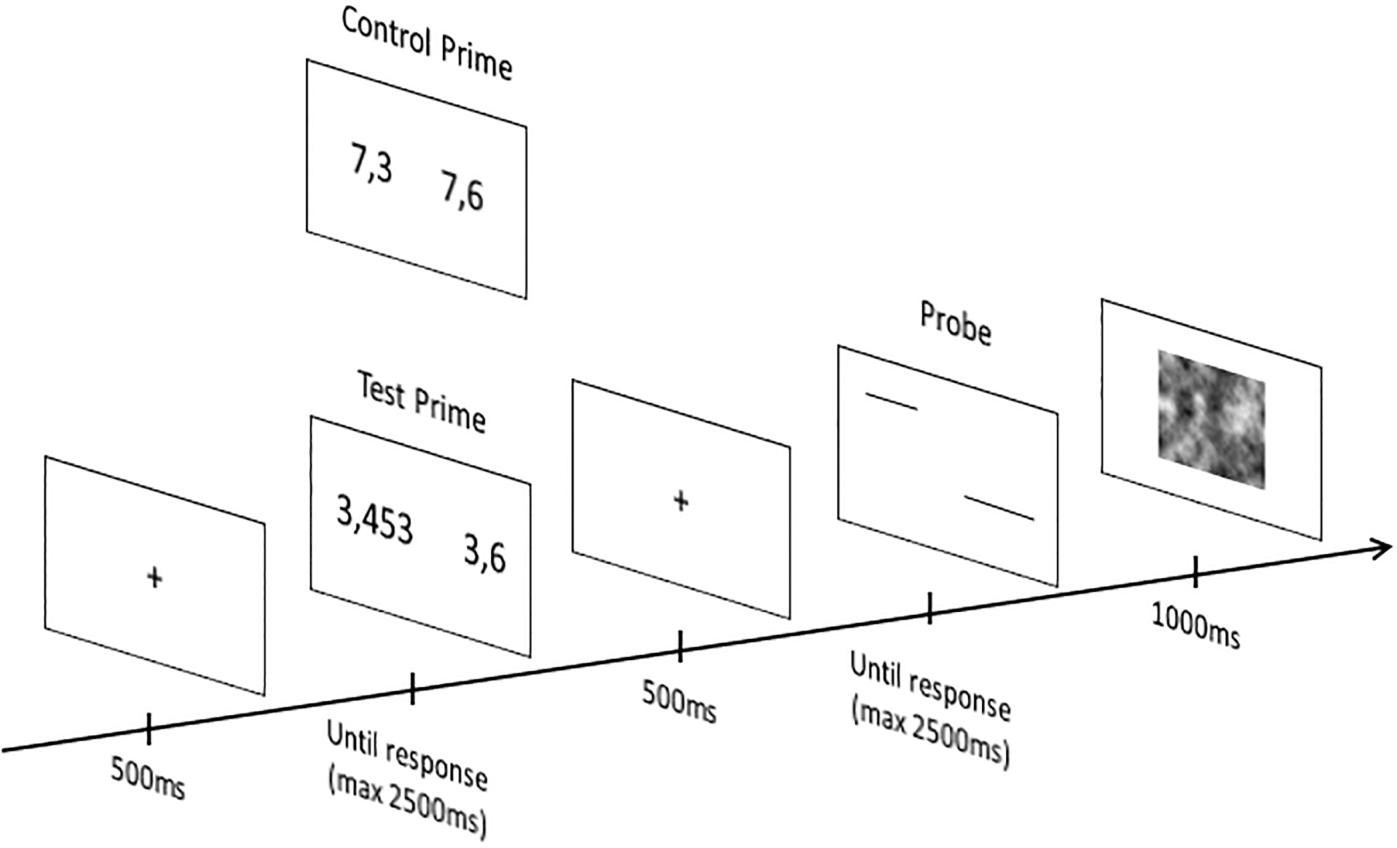
Example of prime and probe items presented in the test and the control conditions. Prime items (i.e., incongruent and neutral items) differed between the two conditions but the probe items were similar.

The materials and procedure were identical to the ones used in Experiment 1 except that probes consisted of pairs of lines: one line appears on the right and one on the left of the centre of the screen at random location. The ratio of lengths between the two lines in a pair was chosen so that the difference in length was easily perceivable. Four different length ratios were used (1.10, 1.2, 1.25, and 1.37) with three levels of lengths (10, 12 and 15 cm), leading to a total of 12 different pairs. Lengths ranged from 7.29 to 18.75 across all 12 pairs. For each trial, children performed two comparison tasks: first on a pair of decimals (i.e., the prime) and then on a pair of lines (i.e., the probe), see Fig 2. On the prime, children judged which of the two decimals was the largest and, on the probe, which of the two lines was the longest by pressing the left or right button of the mouse to indicate that the stimulus presented on the left or the right side of the screen was the largest (for decimal numbers) or longest (for lines). Incongruent items, pairs of decimal numbers in which the smallest decimal had the greatest number of digits after the decimal point (e.g., 3.453 vs. 3.6) served as the prime in the test condition and neutral items, pairs of decimal numbers with the same number of digits after the decimal point (e.g., 7.3 vs. 7.6) served as the prime in the control condition. In both the test and the control conditions, the probe was a pair of lines of different lengths. We also included filler primes consisting of congruent items, decimal pairs where the largest decimal had the greatest number of digits after the decimal point (e.g., 5.644 vs. 5.4) to prevent children from choosing systematically the smallest decimal when the length of the two decimal numbers differed.

Children first performed a block of 5 practice trials with pairs of decimal numbers (1 congruent item, then 2 neutral items and finally 2 incongruent items) and 5 pairs of lines different from the ones used in the experimental trials. Children received simple feedback (correct/incorrect) regarding the correctness of their answers. Then, children performed a block of 60 trials: 24 control trials, 24 test trials and 12 filer trials. All sequences of motor responses between the prime and the probe appeared equally often. The order of the trial was randomized, except no more than three tests or control trials could occur in a row.

#### Results

After deleting outliers defined as in Experiment 1 (M = 10 ± 9%), we averaged the RTs and accuracies separately for control and test primes and probes. As in Experiment 1, we then computed for each child their IES for the primes and the probes of the control and the test conditions. Note that in accordance with the logic of the NP paradigm, we only analysed accuracies, RTs and IES on the probe items preceded by prime items performed correctly. As in Experiment 1, our data complied with the recommendations of Bruyer and Brysbaert [52] for the use of IES: accuracy rate was high (i.e., greater than 95%), accuracies and RTs went in the same direction and we observed no speed-accuracy trade-off (*r*s > .12, *p*s > .45) The IES enables us to simplify our analysis by integrating accuracy scores and RTs in one variable and appropriately weighing the impact of speed and accuracy. For each of the analyses, we report the effect size either in the ANOVA (partial eta squared) or in terms of the difference of the means (Cohen’s d). A Bayesian repeated measure ANOVA and a two-sided Bayesian paired t-test (with a default Cauchy prior width of r = 0.707) were used to quantify the evidence for (BF_01_) or against (BF_10_) the null hypothesis. Bayesian Analyses were performed using the JASP software (Version 0.8.3.1).

A repeated measures Analysis of Variance (ANOVA) on the prime RTs, IES, and accuracies revealed a significant main effect of the type of prime (congruent vs. neutral vs. incongruent items) for RTs, F(2, 72) = 12.81, *p* < .001, *η*_p_^2^ = .26, BF_10_ = 1082 and for IES, F(2, 72) = 13.20, *p* < .001, *η*_p_^2^ = .26, BF_10_ = 1418, but not for accuracies probably due to a ceiling effect, F < 1, BF_01_ = 9 (see Table 2). Post-hoc paired bilateral t-test using a Bonferroni correction on the prime RTs and IES revealed that children were less efficient to compare the magnitude of two decimal numbers in the incongruent items (e.g., 1.45 vs. 1.5) than in the neutral items (e.g., 7.45 vs. 7.55), t(36) = 3.94, *p* < .001, *d* = 0.64, BF_10_ = 78 for RTs and t(36) = 4.01, *p* < .001, *d* = 0.66, BF_10_ = 94 for IES. Children were also less efficient to compare the magnitude of two decimal numbers in the incongruent items (e.g., 1.45 vs. 1.5) than in the congruent trials (e.g., 5.644 vs. 5.4), t(36) = 3.57, *p* = .003, *d* = 0.58, BF_10_ = 30 for RTs and t(36) = 3.60, *p* < .001, *d* = 0.59, BF_10_ = 33 for IES. However, we found no difference in RTs and IES between congruent (e.g., 5.644 vs. 5.4) and neutral (e.g., 7.45 vs. 7.55) items, respectively, t(36) = 1.28, *p* = .62, BF_01_ = 2, for RTs and t(36) = 1.31, *p* = .59, BF_01_ = 2, for IES (See Table 2 and S2 Table).

**Table 2:** Accuracies (%), RTs (ms) and IES in the three types of prime items (incongruent, neutral and congruent items) and the three types of probe items (preceded by an incongruent, neutral or congruent prime item) in Experiment 2. Standard deviations appear in parentheses. Negative priming reflects the difference in performance between incongruent and neutral probe items.

	Prime			Probe			
	Incongruent item	Neutral item	Congruent item	Preceded by an Incongruent prime item	Preceded by a Neutral prime item	Preceded by a Congruent prime item	Negative Priming
Accuracy (%)	98.7 (2.5)	98.7 (3.2)	98.4 (3.8)	92.6 (9.2)	98.3 (3.3)	97.3 (4.4)	5.7 (8.9)
Reaction Time (ms)	1047 (330.7)	886 (275.3)	912 (285.7)	1423 (569.0)	1230 (363.5)	1333 (510.4)	192 (248.7)
IES	1061 (334.6)	898 (334.6)	924.5 (279.0)	1563 (668.4)	1248 (355.6)	1371 (530.9)	314 (362.7)

A repeated measures ANOVA on the probe accuracies, RTs and IES revealed a main effect of the type of probes (i.e., probes preceded by congruent vs. neutral vs. incongruent prime items), F (2, 72) = 10.16, *p* < .001, *η*_p_^2^ = .22, BF_10_ = 467 for accuracies, F(2, 72) = 10.24, *p* < .001, *η*_p_^2^ = .22, BF_10_ = 186 for RTs and F(2, 72) = 15.13, *p* < .001, *η*_p_^2^ = .22, BF_10_ = 4825 for IES. Post-hoc paired bilateral t-test using a Bonferroni correction on the probe accuracies, RTs and IES revealed that children were less efficient to compare the length of two lines after having compared correctly the magnitude of two decimal numbers in the incongruent items (e.g., 1.45 vs. 1.5) than in the neutral items (e.g., 7.45 vs. 7.55), t(36) = 3.92, *p* < .001, *d* = .64, BF_10_ = 74 for accuracies, t(36) = 4.70, *p* < .001, *d* = .77, BF_10_ = 615 for RTs and t(36) = 5.28, *p* < .001, *d* = .86, BF_10_ = 3106 for IES (see Table 2). Similarly, children were less efficient to compare the length of two lines after having compared correctly the magnitude of two decimal numbers in the incongruent items (e.g., 1.45 vs. 1.5) than in the congruent items (e.g., 5.644 vs. 5.4), t(36) = 2.93, *p* = .018, *d* = 0.33, BF_10_ = 6 for IES and t(36) = 2.85, *p* = .02 *d* = 0.47, BF_10_ = 5 for accuracies. However, no significant differences was found for RTs, t(36) = 2.04, p = .14, BF_01_ = 0.8 (see Table 2). Finally, we found no differences on children’s performance between probes preceded by congruent items (e.g., 5.644 vs. 5.4) and probes preceded by neutral items (e.g., 7.45 vs. 7.55), t(36) = 1.24, *p* = .66, BF_01_ = 2, for accuracies and t(36) = 2.39, *p* = .39, BF_01_ = 0.4, for RTs, except for IES, t(36) = 2.64, *p* = .03, *d* = 0.43, BF_10_ = 3 (see Table 2).

### Discussion

As in Experiment 1 and consistent with previous studies [3,12,14,15,16,24], we found that children were less efficient to compare the magnitude of two decimal numbers in incongruent items, when the smallest decimal had the greatest number of digits after the decimal point (e.g., 1.45 vs. 1.5), than in neutral items, when two decimal numbers with the same decimal place (e.g., 7.45 vs. 7.55). Additionally, children were also found to be less efficient to compare two decimal numbers in incongruent items (e.g. 1.45 vs. 1.5) than in congruent items, decimal pairs where the largest decimal had the greatest number of digits after the decimal point (e.g., 5.644 vs. 5.4). Importantly, children were less efficient in comparing the length of two lines after having compared the magnitude of two decimal numbers in incongruent items (e.g., 1.45 vs. 1.5, a context that requires to inhibit the length of the numbers to compare their magnitude) than after having compared the magnitude of two decimal in neutral items, (e.g., 7.45 vs. 7.55, a context in which length does not interferes or facilitate the magnitude comparison). Moreover, children were less efficient in comparing the length of two lines after having compared the magnitude of two decimal numbers in incongruent items (e.g., 1.45 vs. 1.5) than after having compared the magnitude of two decimal in congruent items (e.g., 5.644 vs. 5.4). Taken together the results of Experiment 2 suggest that the difficulty in comparing the magnitude of decimal numbers may be in part due to the difficulty to inhibit the length of the number when it interferes with its magnitude such as in a context in which the smallest decimal number has the largest number of digits (e.g., 1.45 vs. 1.5). We suspect that the overlap of the network of neurons in the IPS involved in the processing of symbolic numbers and numerosity and the neurons coding for nonsymbolic continuous magnitude such as size and length [61,62,63,67] might be at the root of the difficulty to compare the magnitude of decimal numbers when their length interferes with their magnitude.

Note that we found no difference in children’s response to congruent and neutral items or to probe items preceded by congruent and neutral prime items. The lack of difference found between neutral and congruent items—which one would expect if individuals spontaneously focus on the length of the number per se to compare the magnitude of decimal numbers—may be due to the fact that congruent items were used as fillers and thus presented less often than other items (congruent items were presented half as often as neutral and incongruent items: 12 times vs. 24 times) leading to a potential oddball effect [68].

## General discussion

The current study aimed to determine (a) whether 7^th^ grade children must inhibit the “greater the number of digits the greater its magnitude” strategy to correctly compare the magnitude of decimal numbers in which the smallest decimal number has the largest number of digits (1.45 vs. 1.5) and (b) whether the difficulty in comparing decimal numbers in that context could be also due to the fact that the smallest number (1.45) is longer (in regards to its length in a spatial continuous magnitude sense) than the largest one (1.5).

Consistent with previous studies [3,12,14,15,16,24], we found in Experiment 1 and 2 that 7^th^ graders were less efficient to compare the magnitude of two decimal numbers when the smallest one has the greatest number of digits (1.45 vs. 1.5) than when the two decimal numbers have the same number of decimal place (1.8 vs.1.9).

In Experiment 1, children were less efficient to compare the magnitude of two decimal numbers in which the largest one had the greatest number of digits (e.g., 1.345 vs. 1.2) after comparing the magnitude of two digits in which the smallest one had the greatest number of digits (1.45 vs. 1.5) than after comparing two decimal numbers with the same number of decimal place (1.8 vs.1.9). This typical negative priming effect suggests that children must inhibit the “greater the number of digits the greater its magnitude” misconception when comparing the magnitude of decimal numbers in which the smallest decimal number has the greatest number of digits (1.45 vs. 1.5). This misconception is probably a consequence of an overgeneralization of children’s knowledge of whole numbers to decimal numbers [4,20,69] and in particular children’s knowledge that numbers with more digits are larger [54]. Our finding fits well with conceptual change theories arguing that a correct understanding of rational decimal numbers may coexist with an earlier intuitive understanding of whole numbers [15]. Previous studies have actually demonstrated that educated adults and mathematical experts still show signs of whole number bias on a variety of rational number tasks [12, 70,71].

In Experiment 2, we found that 7^th^ graders were less efficient to compare the length of two lines after comparing the magnitude of decimal numbers in which the smallest decimal number has the greatest number of digits (1.45 vs. 1.5) than after comparing the magnitude of two decimal numbers with the same number of decimal place (1.8 vs.1.9). The negative priming effect reported in Experiment 2 suggests that comparing the magnitude of decimal numbers in which the smallest decimal number has the greatest number of digits (1.45 vs. 1.5) might require to inhibit the length of the decimal number per se in addition to inhibit the “greater the number of digits the greater its magnitude” misconception.

We suspect that inhibiting the length of the decimal number to process its magnitude in this context might be needed because neurons in the IPS involved in the processing of symbolic numbers and numerosity overlap with the neurons coding for non-symbolic continuous magnitude such as length [61,62,63].

The overlap of the functional networks involved in symbolic dimension of magnitude and non-symbolic dimensions of magnitude such as length could be a consequence of the neuronal recycling induced by learning mathematics. According to the neuronal recycling theory, cultural tools such as reading and mathematics are too recent to have had an impact on the human genome and thus these cultural tools rely on a process of neuronal recycling according to which pre-existing brain circuitry are recycled to carry-out these new functions [72]. Mathematical learning in particular would induce recycling neurons of the IPS, originally dedicated to the processing of continuous non-symbolic dimensions of magnitude to process symbolic and non-symbolic discrete dimension of magnitude [59]. Pre-existing properties of the neurons being recycled could induce errors such as those observed in decimal comparison, and inhibitory control might be a core mechanism allowing correcting errors induced by the neuronal recycling process. This assumption is coherent with findings that overcoming systematic errors in reading and mirror error in particular (confusing ‘b’ for ‘d’) depended upon the capacity to inhibit the original function (here the mirror generalization process) of the neurons recycled [41,73].

Note that while both experiments demonstrate that one needs to inhibit the whole number bias to compare the magnitude of decimal numbers in which the smallest decimal number has the largest number of digits (i.e., incongruent items), they allowed us to determine the nature of the cognitive constructs that were inhibited. Thus, the two experiments provide evidence that the whole number bias is rooted in part on the number of digits after the decimal points (i.e., the “greater the number of digits the greater its magnitude”, see Experiment 1) and in part on the length of the decimal number per se (i.e., see Experiment 2).

One could argue that the negative priming effect observed on the congruent probe items between the control and test conditions might be essentially driven by the difference in the difficulty between the incongruent items and the neutral items presented on the primes respectively in the test and control conditions. However, previous studies have provided evidence that the negative priming effect is not a by-product of performing easier vs. more difficult items on the prime [74,75,76]. For instance, negative priming effects are classically reported in the Stroop task in which incongruent items are presented on the prime in both the test and the control conditions. In these paradigms, a negative priming effect is observed when participants require more time to identify the ink color (i.e., the task-relevant information) in an incongruent Stroop item (e.g., BLUE printed in red) when the ink color is the color denoted by the word (i.e., the task-irrelevant information to inhibit) in the preceding incongruent Stroop item (e.g., RED printed in green) than when the ink color is not the color denoted by the word in the preceding incongruent Stroop item (e.g., YELLOW printed in green).

Moreover, one could argue that a conflict adaptation paradigm might have provided better evidence that inhibitory control is needed to compare the magnitude of decimal numbers in which the smallest decimal number has the greatest number of digits after the decimal point. However, conflict adaptation does not provide evidence per se that inhibitory control is required in a given task, it allows to investigate that cognitive control can be sustained from one item to the next [77,78]. In addition, conflict adaptation tasks are dependent on the ability to engage proactive control, which is still developing between 8 and 10 years old [79] and might interact with conflict adaptation effects [80]. Finally, as opposed to the conflict adaptation paradigm, the negative priming paradigm allows to determine the nature of the strategy, bias, or misconception that must be inhibited which is one of the goals of the present study [37,41,46,64,73].

Despite being obtained in laboratory experiments, our findings have important educational implications. First, in light of the lack of awareness of teachers about the role played by inhibitory control in fundamental academic learning [81], it seems critical to raise their awareness of the importance of inhibition in decimal magnitude comparison. A consequence of this lack of awareness in the context of decimal magnitude comparison is that teachers might interpret students’ errors as revealing a misunderstanding of the mathematical principles governing the comparison of decimal numbers, whilst these errors might actually reveal a difficulty to inhibit the “greater the number of digits the greater its magnitude” misconception, as suggested by the present study.

Second, considering that failure to inhibit the “greater the number of digits the greater its magnitude” misconception might be at the root of systematic errors when comparing decimal numbers in which the smallest decimal number has the largest number of digits (1.45 vs. 1.5), pedagogical interventions based solely on learning the mathematical principles governing the comparison of decimal numbers might not be sufficient to overcome these systematic errors. Therefore, pedagogical interventions based on metacognitive executive (inhibitory control) learning could provide a more effective way to help children overcome systematic difficulties when comparing decimal numbers in which the smallest decimal number has the largest number of digits (1.45 vs. 1.5). Metacognitive inhibitory control intervention typically consists in emphasizing both the logico-mathematical principles to use and the misconception to inhibit to avoid systematic errors when solving a problem. Several studies provided converging evidence that this type of intervention is more effective in overcoming systematic logico-mathematical reasoning errors than more classical interventions emphasizing only the logico-mathematical principles to use [28,82,83]. In the context of learning to compare decimal numbers, a metacognitive inhibitory control intervention would typically emphasise the need to inhibit the “greater the number of digits the greater its magnitude” misconception and the need to activate the mathematical principles governing the comparison of decimal numbers (i.e., comparing the decimal numbers by comparing the magnitude of the different place values starting from the first one after the decimal point).

Although no study to date has tested the effectiveness of such metacognitive inhibitory control intervention in the context of decimal number comparison, findings from one previous study suggest that raising children’s awareness of the “greater the number of digits the greater its magnitude” misconception (by presenting correct and incorrect examples) can already improve their understanding of the mathematical principles governing decimal number magnitude [24] see also [84] for similar evidence in other domains. Thus, it is likely that raising children’s awareness of this misconception and explicitly teaching them to inhibit this misconception when comparing decimal numbers in which the smallest decimal number has the largest number of digits (1.45 vs. 1.5) could be effective to overcome systematic errors in this context.

In conclusion, our results further suggest that inhibitory control is one of the core mechanisms at the root of mathematical development [21,32,33,34,35,36,37,38,85] and more generally of cognitive development [26,27,28,29,30,31].

## Supporting information

S1 Table Individual accuracy rates, response times and inverse efficiency scores (IES) for the prime and the probe in the test and control conditions in Experiment 1. (XLSX)

S2 Table Individual accuracy rates, response times and inverse efficiency scores (IES) for the prime and the probe in the test, control and filler conditions in Experiment 2. (XLSX)
